# Harnessing the potential of blood donation archives for influenza surveillance and control

**DOI:** 10.1371/journal.pone.0233605

**Published:** 2020-05-29

**Authors:** Yanyu Zhang, Kathy Leung, Ranawaka A. P. M. Perera, Cheuk-Kwong Lee, J. S. Malik Peiris, Joseph T. Wu

**Affiliations:** 1 School of Public Health, WHO Collaborating Center for Infectious Disease Epidemiology and Control, The University of Hong Kong, Hong Kong Special Administrative Region, China; 2 Center of Influenza Research and School of Public Health, The University of Hong Kong, Hong Kong Special Administrative Region, China; 3 Hong Kong Red Cross Blood Transfusion Service, Hospital Authority, Hong Kong Special Administrative Region, China; University of South Dakota, UNITED STATES

## Abstract

Many blood donation services around the globe maintain large archives of serum and/or plasma specimens of blood donations which could potentially be used for serologic surveillance and risk assessment of influenza. Harnessing this potential requires robust evidence that the outcomes of influenza serology in plasma, which is rarely used, is consistent with that in serum, which is the conventional choice of specimens for influenza serology. We harvested EDTA-plasma specimens from the blood donation archives of Hong Kong Red Cross Transfusion Services, where EDTA is the type of anticoagulant used for plasma collection, compared their antibody titers and responses to that in serum. Influenza A/H1N1/California/7/2009 and A/H3N2/Victoria/208/2009 were the test strains. Our results showed that antibody titers in 609 matched serum/EDTA-plasma specimens (i.e. obtained from the same donor at the same time) had good agreement inferred by Intraclass Correlation Coefficient, the value of which was 0.82 (95% CI: 0.77–0.86) for hemagglutination inhibition assay and 0.95 (95% CI: 0.93–0.96) for microneutralization assay; seroconversion rates (based on hemagglutination inhibition titers) during the 2010 and 2011 influenza seasons in Hong Kong inferred from paired EDTA-plasma were similar to that inferred from paired sera. Our study provided the proof-of-concept that blood donation archives could be leveraged as a valuable source of longitudinal blood specimens for the surveillance, control and risk assessment of both pandemic and seasonal influenza.

## Introduction

Influenza is a major global health threat [1]. Serology is often regarded as the most reliable method for inferring previous exposure to and immunity against influenza [2]. As such, influenza seroepidemiology can provide timely and accurate estimates of incidence and severity to informing decisions regarding the scale and targeting of response for both pandemics and seasonal epidemics [3, 4].

Blood donation services in many populations (e.g. the United Kingdom, Australia, France) archive serum and/or plasma specimens from each blood donation to monitor blood safety and assist retrospective studies of transfusion-transmitted infections of pathogens such as hepatitis C and human immunodeficiency virus [5]. The length of specimen storage in these archives is typically 1–5 years (e.g. in Australia, England, France, and the Netherlands) but can be as long as 30 years (in Japan) or even indefinite (in Scotland) [5]. We postulate that these large blood archives could provide a valuable source of longitudinal specimens for influenza serosurveillance if antibody titers and responses measured with plasma and serum are consistent.

Conventionally, serum has been the choice of specimen for influenza serology, and hemagglutination inhibition (HI) and microneutralization (MN) assays are the two most common assays for measuring antibody titers and responses against influenza. The choice between serum and plasma is largely determined by the requirements of specific assays or preferences of different laboratories. Although plasma has recently been used in several influenza serologic studies [6–12], these studies have provided little or no documentation on the validity of plasma serology. Anticoagulants (citrate, heparin or EDTA) in plasma specimens can interfere with antibody-antigen reactions and may inhibit the activity of some enzyme reagents [13]. Defang et al. compared the HI antibody titers of serum and plasma (which contained either heparin or citrate) collected from the same subjects at the same time against five strains of influenza [14]. They reported that titers in serum and plasma were highly correlated and that seroconversion rates were largely unaffected by specimen type. Although their results suggested that citrated and heparinized plasma could be used in HI assays for the five influenza strains tested, the validity of plasma serology with EDTA, which has been regarded as a better anticoagulant than heparin or citrate [15], and other influenza strains remains unknown.

In summary, there is a lack of evidence on the validity of plasma influenza serology, especially for EDTA-plasma. Particularly, EDTA-plasma is the choice of blood specimens preferred by biobanks [16, 17] (e.g. the UK Biobank [18]) and archived by Hong Kong Red Cross Blood Transfusion Service, because it is available for a wide range of DNA-based and protein assays. Therefore, the objective of this study was to build an evidence base for the validity of EDTA-plasma influenza serology and provide the proof-of-concept that specimens from blood donation archives can be harnessed for influenza surveillance and risk assessment.

## Materials and methods

### Sources of specimens

**Fig 1:**
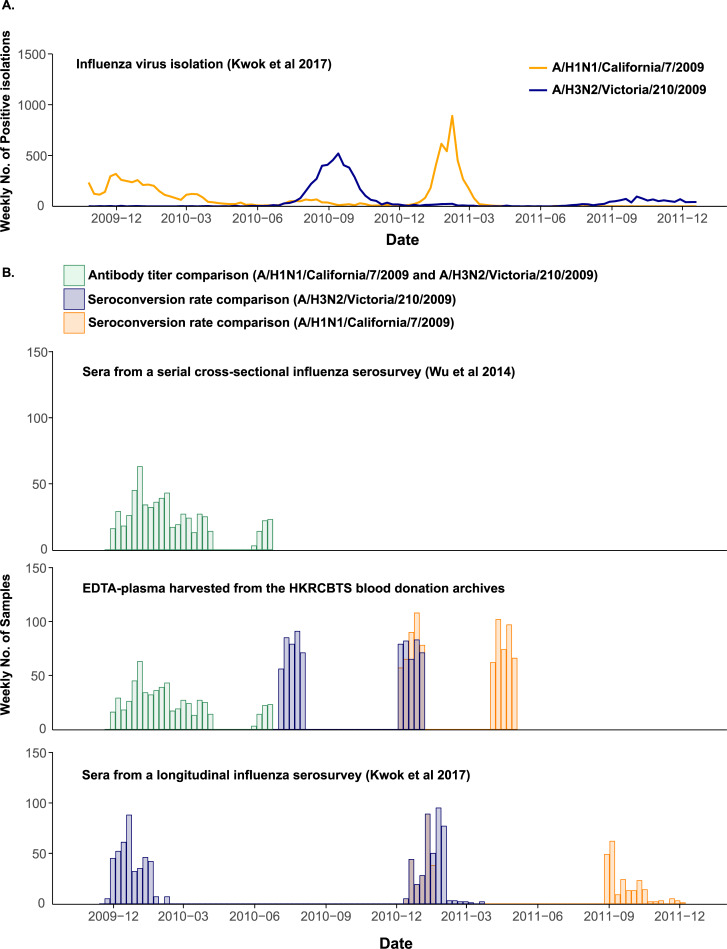
Influenza activity in Hong Kong during 2009–2011 and the sources of specimens for comparative analyses. (A) Influenza virus isolation [20]; (B) Specimens included sera from a serial cross-sectional influenza serosurvey [3], EDTA-plasma harvested from the HKRCBTS blood donation archives, and sera from a longitudinal influenza serosurvey [20]. Color codes indicate the specimens used for the different comparative analyses in this study: green indicates 609 matched serum/EDTA-plasma specimens for antibody titer comparison; purple indicates 340 paired sera and 376 paired EDTA-plasma for estimating seroconversion rate against A/H3N2 during the 2010 season; and orange indicates 190 paired sera and 392 paired EDTA-plasma for estimating seroconversion rate against A/H1N1 during the 2011 season.

Our group conducted a serial cross-sectional serosurvey based on 14,000 sera from blood donors taken when the 2009 pandemic H1N1 began circulating in Hong Kong [19]. From 21 June to 31 December 2009, blood donors from the four largest donation centres (Mongkok, Causeway bay, Kwun Tong, and Tsuen Wan) of Hong Kong Red Cross Blood Transfusion Service (HKRCBTS) were invited to participate in our serological surveillance study. When they donated their blood, they were asked to provide one extra serum sample that stored at -20°C in the Li Ka Shing Faculty of Medicine. To monitor blood safety, the HKRCBTS archives one 5 ml EDTA-plasma specimen from each blood donation for 15 months. We harvested the expired specimens from the donation archive that were donated during December 2009 to December 2011. Specifically, these expired specimens included 10,972 longitudinal EDTA-plasma specimens from 2,331 repeated donors who donated their blood between December 2009 and December 2011. In analysis of antibody titer comparison, we matched 609 EDTA-plasma specimens that expired from the blood donation archive with sera collected simultaneously from the same donors between December 2009 and June 2010 (Fig 1).

To validate the feasibility of EDTA-plasma specimens for influenza incidence estimate, we compared seroconversion rates of influenza epidemics using paired EDTA-plasma from the same donor to those derived from paired sera in an independent longitudinal serological study by Kwok et al. [20]. Participants in Kwok et al. were recruited via random digit dialling of household landlines, asked to attend the study clinic and answer a questionnaire and to provide a 5 ml serum sample. Also, the responding individuals were asked to invite other eligible members within the household including children. It was initiated with the first recruitment round (Round 1) between July and September 2009. Then, they invited participants to return to the clinic during November 2009–February 2010 (Round 2), December 2010–March 2011 (Round 3) and August–December 2011 (Round 4).

Two epidemics happened in this study period in Hong Kong: seasonal A/H3N2 during July–December 2010 and pandemic A/H1N1 during January–March 2011. We used samples donated in July and December 2010 as the pre- and post-epidemic samples for the seasonal A/H3N2 wave. In the study by Kwok et al. [20], Round 2 and Round 3 sampling bracketed the 2010 seasonal A/H3N2 wave, thus we used samples obtained in Rounds 2 and Round 3 as pre- and post-epidemic samples. In total, 376 blood donors donated plasma samples twice while 340 participants provided paired sera in the study by Kwok et al. HI seroconversion rates against A/H3N2/Victoria/210/2009 (A/H3N2) were estimated by using 376 paired EDTA-plasma and 340 paired sera from Kwok et al. (data is publicly available).

Similarly, we used samples donated in December 2010 and April 2011 as the pre- and post-epidemic samples for the seasonal A/H1N1 wave. Although Round 2 and Round 4 in Kwok et al. study bracketed the 2011 pandemic A/H1N1 wave, this period also included the 2010 seasonal A/H3N2 wave. In addition, the interval between Round 2 and Round 4 was long so that it might underestimate infection attack rates due to the potential waning immunity. Therefore, Round 3 and Round 4 sampling were used as the pre- and post-epidemic of A/H1H1 for Kwok et al. study. In order to reduce the bias in pre-epidemic sampling, December 2010–15 January 2011 was defined as the pre-epidemic of A/H1N1 in the study by Kwok et al. Thus, 392 donors provided paired EDTA-plasma from our samples and 190 participants had paired sera from Kwok et al during this time period, which were used for estimating HI seroconversion rates against A/H1N1/California/7/2009 (A/H1N1).

The age range of participants in Kwok et al study was from 5 to 80 years old, whereas the age of blood donors was between 16–65 years old. Those individuals aged < 16 and > 65 years old were removed for estimating age-standardization seroconversion rates. Totally, 340 participants for A/H3N2 and 190 participants for A/H1N1 from Kwok et al study were included for calculation.

The study was approved by Institutional Review Board of The University of Hong Kong/ Hospital Authority Hong Kong West Cluster (HKU/HA HKW IRB). The reference number is UW 17–213. All methods were performed in accordance with relevant guidelines and regulations.

### Serological assays

The protocol of HI and MN assays were in accordance with the Manual for the Laboratory Diagnosis and Virological Surveillance of Influenza by World Health Organization [21]. The details of the two assays are documented in S1 File.

### Statistical analysis

Titers < 1:10 were assigned a value of 1:5 for computational purposes. When comparing titers in matched serum/EDTA-plasma specimens, we used the Stuart-Maxwell test to assess marginal homogeneity with 0.05 as the p-value threshold for statistical significance [22, 23]. If the Stuart-Maxwell test indicated a statistically significant lack of marginal homogeneity, we regarded antibody titer distribution in plasma to be inconsistent with that in serum.

Although statistical significance is concerned with whether the measured effect is real, it does not imply that the result has any influence. Effect size can quantify the difference between two groups and emphasize the magnitude of difference [24, 25]. Its value represents the practical significance of the findings in the context of the study hypothesis independent of sample size. Therefore, the effect size was used to measure the strength of difference in antibody titer distributions between matched serum and EDTA-plasma. Cohen’s *d* is one of the common methods to measure effect size; the interpretation guidelines for Cohen’s *d* are as follows [26]: an effect size of 0.2, 0.5 and 0.8 corresponds to a small, medium and large level, respectively.

Furthermore, we quantified the degree of agreement between the titers in matched serum/EDTA-plasma specimens using intraclass correlation coefficient (ICC) [27, 28]. Specifically, we calculated ICC using the two-way mixed effect, consistency/absolute agreement, single measurement model. As the relationship of antibody titers to matched specimens was the concern of this study, both definitions of consistency and absolute agreement were used to calculate the ICC values. The results of the ICC analysis range between 0.0 and 1.0, with values closer to 1.0 representing stronger reliability. The guideline of interpretation of the ICC value are as follows [29]: less than 0.5, between 0.5 and 0.75, between 0.75 and 0.9, and greater than 0.9 indicates poor, moderate, good, and excellent reliability, respectively.

We used Bayesian inference with beta conjugates to estimate the seroconversion rates in each age group [30]. Specifically, we assumed non-informative flat priors, i.e. all priors were beta distributions with parameters *α* = 1 and *β* = 1. Under these assumptions, if there were *x* seroconversions among *n* paired specimens, the posterior distribution of the seroconversion rate would be a beta distribution with parameters *α* = 1+*x* and *β* = 1+*n*−*x* [31, 32]. When estimating the age-standardised seroconversion rates from paired sera and paired EDTA-plasma, we stratified the subjects into the following age groups: 16–25, 26–35, 36–45, 46–55 and 56–65. If *y_i_* was the seroconversion rate for age group *i*, then the age-standardised seroconversion rate was simply ∑*_i_y_i_N_i_*/∑*_i_N_i_*, where *N_i_* corresponds to the Hong Kong age distributions obtained from the Census and Statistics Department [33]. All statistics were performed using R version 3.6.1 (R Foundation for Statistical Computing, Vienna, Austria).

## Results

### Comparing antibody titers in matched serum/EDTA-plasma specimens

We compared the HI and MN antibody titers in these matched serum/EDTA-plasma specimens against A/H1N1 and A/H3N2, which caused the influenza epidemics in Hong Kong during 2009–2011. Conventionally, titers within one dilution factor (i.e. two-fold difference) are regarded as identical to account for inter-laboratory and intra-experiment errors [34]. Among the 609 matched serum/EDTA-plasma specimens, 92.3% (562/609) and 89.2% (543/609) had HI titers within a two-fold difference for A/H1N1 and A/H3N2, respectively. In particular, seven matched serum/EDTA-plasma specimens had more than a two-fold difference for both strains.

**Fig 2:**
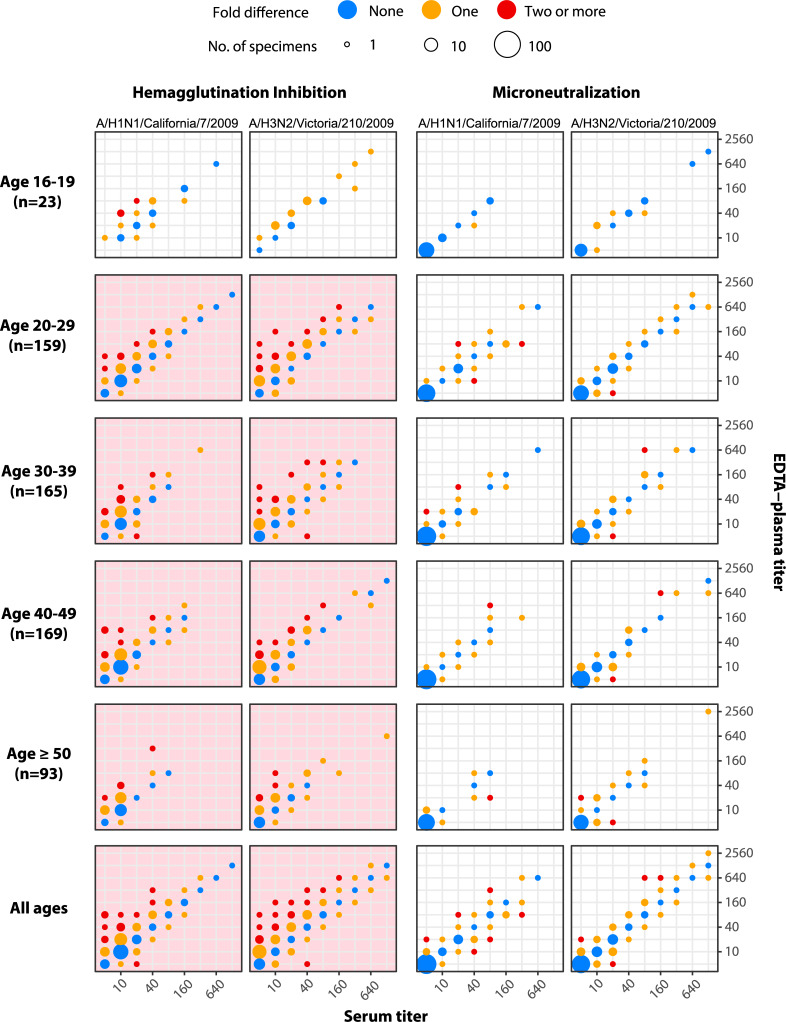
Comparison of antibody titers in 609 matched serum/EDTA-plasma specimens. Serum and EDTA-plasma specimens were collected from the same donor at the same time during 2009 (Fig 1). Color codes indicate the fold difference in antibody titers between the matched serum/EDTA-plasma specimens: blue, orange and red indicate no, one-fold and two-fold or more difference, respectively. Pink shades indicate p<0.05 in Stuart-Maxwell test (i.e. statistically significant lack of marginal homogeneity between serum and EDTA-plasma titer distributions).

The difference in HI titer distributions between serum and EDTA-plasma was statistically significant (p < 0.05, Stuart-Maxwell test, pink shade in Fig 2) for both strains and nearly all age groups except for the 16–19 age group (probably due to the limited sample size). Nonetheless, the difference was practically insignificant as it showed a small level of effect size, the Cohen’s *d* value for the difference of HI titer distributions was 0.42 (95% CI: 0.38–0.47) in A/H1N1 and 0.4 (95% CI: 0.35–0.44) in A/H3N2. The results of the ICC analysis suggested that titers in EDTA-plasma could be equal to titers in serum plus a systematic error/difference, rather than having absolute agreement in HI titers between matched specimens (Table 1). Therefore, systematic errors/differences exist in the detection of antibody titers in EDTA-plasma when performing HI assays.

**Table 1:** ICC values of HI titers in all pairs under the definition of absolute agreement *versus* consistency.

ICC analysis of HI titers in matched pairs				
Age (N)	A/H1N1		A/H3N2	
	Absolute agreement (95% CI)	Consistency (95% CI)	Absolute agreement (95% CI)	Consistency (95% CI)
16–19 (23)	0.84 (0.66–0.95)	0.86 (0.69–0.95)	0.93 (0.88–0.96)	0.96 (0.92–0.98)
20–29 (159)	0.85 (0.78–0.9)	0.9 (0.84–0.93)	0.79 (0.72–0.85)	0.86 (0.81–0.9)
30–39 (165)	0.59 (0.45–0.73)	0.69 (0.55–0.81)	0.74 (0.64–0.81)	0.8 (0.71–0.86)
40–49 (169)	0.68 (0.53–0.78)	0.75 (0.61–0.84)	0.78 (0.68–0.86)	0.85 (0.78–0.91)
50–69 (93)	0.59 (0.43–0.72)	0.71 (0.55–0.81)	0.77 (0.62–0.88)	0.82 (0.71–0.9)
Total	0.75 (0.69–0.8)	0.82 (0.77–0.86)	0.8 (0.76–0.83)	0.86 (0.83–0.89)

95% CI, 95% confidence interval.

Compared to HI titers, MN titers had stronger agreement among the matched specimens. For both strains, only 1.3% (8/609) of the matched serum/EDTA-plasma specimens had more than a two-fold difference in MN titers, and only one EDTA-plasma specimen had more than a two-fold difference in MN titers. There was no statistically significant difference in MN titer distributions between the serum and EDTA-plasma specimens (p > 0.05, Stuart-Maxwell test, Fig 2). The ICCs were uniformly above 0.91 for both absolute agreement and consistency (Table 2), indicating excellent agreement in MN titer readings within matched specimens.

**Table 2:** ICC values of MN titers in all pairs under the definition of absolute agreement *versus* consistency.

ICC analysis of MN titers in matched pairs				
Age (N)	A/H1N1		A/H3N2	
	Absolute agreement (95% CI)	Consistency (95% CI)	Absolute agreement (95% CI)	Consistency (95% CI)
16–19 (23)	0.99 (0.93–1)	0.99 (0.93–1)	0.97 (0.92–0.99)	0.97 (0.91–0.99)
20–29 (159)	0.95 (0.92–0.97)	0.95 (0.92–0.97)	0.96 (0.94–0.98)	0.96 (0.94–0.98)
30–39 (165)	0.94 (0.87–0.97)	0.94 (0.87–0.97)	0.93 (0.89–0.96)	0.93 (0.89–0.96)
40–49 (169)	0.95 (0.92–0.97)	0.95 (0.92–0.97)	0.96 (0.93–0.98)	0.96 (0.93–0.97)
50–69 (93)	0.91 (0.78–0.98)	0.91 (0.78–0.98)	0.94 (0.87–0.97)	0.94 (0.86–0.97)
Total	0.95 (0.93–0.96)	0.95 (0.93–0.96)	0.95 (0.94–0.96)	0.95 (0.94–0.96)

95% CI, 95% confidence interval.

### Comparing seroconversion rates in paired sera and paired EDTA-plasma during influenza seasons

There were two influenza epidemics in Hong Kong during 2010–2011: an A/H3N2 epidemic in July–December 2010; and an A/H1N1 epidemic in January–March 2011 (Fig 1). From July 2009 to December 2011, Kwok et al. ran a cohort study in Hong Kong to estimate the incidence of influenza for both epidemics based on HI seroconversion rates in paired sera [20].

We estimated the age-standardised (for the population aged 16–65) HI seroconversion rate in the 2010 A/H3N2 epidemic using 340 paired sera from Kwok et al. and 376 paired EDTA-plasma from the HKRCBTS archives (Fig 1). Under the conventional definition of seroconversion (i.e. a four-fold or greater rise in antibody titers and the latter titer is at least ≥ 1:40), the age-standardised seroconversion rates estimated from the paired sera and paired EDTA-plasma were 10% (7–14%) and 13% (10–17%), respectively. Similarly, we estimated the age-standardised HI seroconversion rate in the 2011 A/H1N1 epidemic using 190 paired sera from Kwok et al. and 392 paired EDTA-plasma from the HKRCBTS archives. Under the conventional definition of seroconversion, the age-standardized seroconversion rates estimated from the paired sera and paired EDTA-plasma were 18% (12–25%) and 17% (14–22%), respectively.

**Fig 3:**
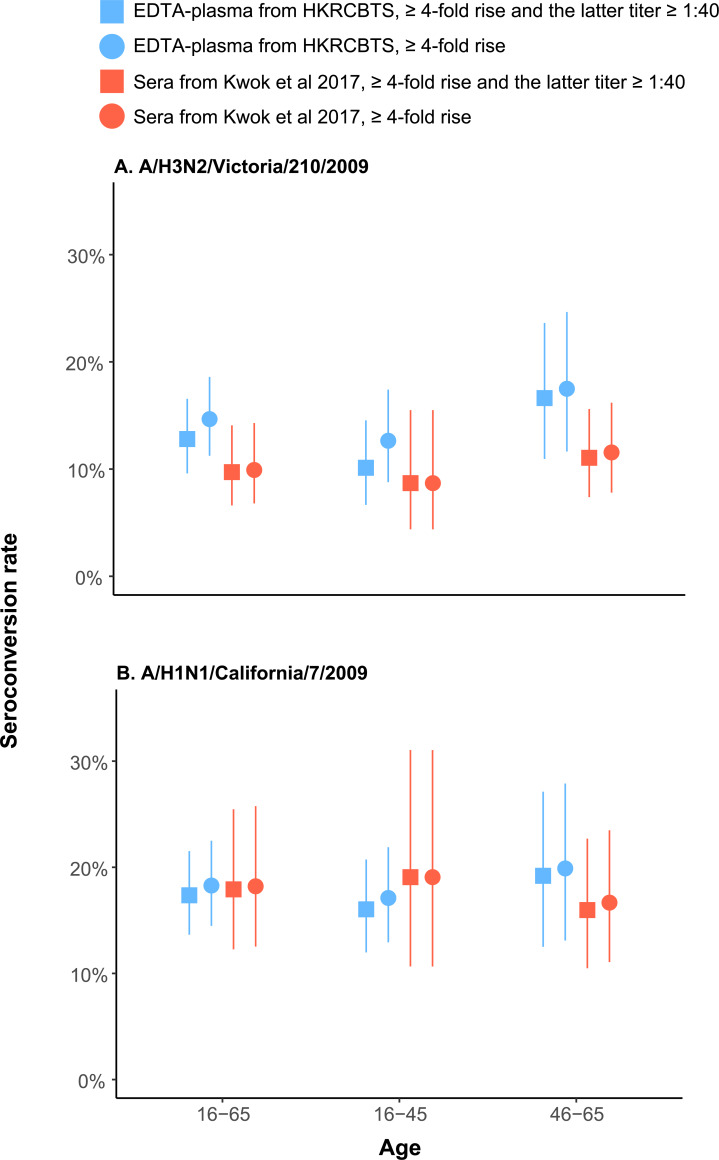
Comparison of the age-standardized HI seroconversion rates in paired sera and paired EDTA-plasma. (A) The 2010 H3N2 influenza season in Hong Kong caused by A/H3N2/Victoria/210/2009. (B) The 2011 H1N1 influenza season in Hong Kong caused by A/H1N1/California/7/2009. Color codes indicate the source of paired specimens used for calculating seroconversion rates: blue indicates paired EDTA plasma from HKRCBTS blood donation archives, while red indicates paired sera from Kwok et al. [20]. Square and circle indicate the conventional (≥4-fold rise and the latter titer ≥1:40) and alternative (≥4-fold rise) definition of seroconversion, vertical bars indicate 95% confidence intervals.

The age-standardised seroconversion rates estimated from paired sera and paired EDTA-plasma remained similar when the subjects were stratified into two age groups with 45 years old as the age cut-off (Fig 3). As a recent study suggested that the convalescent HI titer of some infections may not reach 1:40 [35], we also considered a four-fold or greater rise in antibody titers as an alternative definition for seroconversion (i.e. the latter titer can be 1:20). The age-standardised seroconversion rates remained similar under this definition (Fig 3).

## Discussion

This study is the first to extensively validate the use of EDTA-plasma for influenza serology. Our results suggested that antibody titers and responses against two influenza strains in EDTA-plasma are largely consistent with that in sera, especially when MN assay is used. Since the excellent agreement of MN titers was present in both matched pairs and the whole titer distributions, EDTA-plasma should be substituted for serum in MN assays. While HI titers in matched specimens were correlated in an additive manner, i.e. the HI titer in EDTA-plasma could equal that in serum plus a systematic error/difference. The systematic errors/differences might come from two sources: (i) the difference between serum and plasma. Plasma is the liquid part of blood, the whole blood after removing red blood cells, white blood cells and platelets through centrifuging. Particularly, plasma is prevented from clotting with an anticoagulant (such as sodium citrate or EDTA). Serum is the liquid part of coagulated (clotted) blood, and it is free from clotting proteins but contains clotting metabolites as a result of a clotting process. Therefore, plasma includes the clotting factors because the blood is prevented from clotting, while serum is the liquid part after the blood is allowed to clot and separate; (ii) the differences between HI and MN assay. HI is the assay for detection of antibodies to influenza viruses, which block sialic acid receptors on red blood cells (RBCs) binding to hemagglutinin glycoprotein (HA) on surface of influenza viruses. The main goal of HI assay is to characterize the concentration of antibodies in blood samples (e.g. serum, plasma). Before performing HI assay, blood samples must be treated with receptor-destroying enzyme (RDE) to remove the various sialic acid-containing glycans in samples, which may bind to HA resulting in a false positive reading. In addition, some samples must also be absorbed with RBCs to remove non-specific agglutinins of RBCs to prevent false-negative results. While MN assay is a highly sensitive and specific method for detecting strain-specific antibodies that block virus entry into MDCK cells. It is unknown that whether anticoagulant or other clotting factors that present in plasma might have interaction with RDE enzyme, which could cause a false positive result. Compare to HI assay, MN assay can show consistent results as the virus-antibody binding is more stable. However, a recent study by Perera et al [36] regarding serological assay for severe acute respiratory syndrome coronavirus (SARS-CoV-2) demonstrated that MN titer in heparinised plasma is higher than that detected in serum from the same patient. Therefore, the inconsistency in HI titer distributions should be practically insignificant as these differences might be caused by systematic errors/differences.

Nevertheless, the consistency of antibody titers between matched serum and EDTA-plasma could be attributed to the large proportion of specimens that were seronegative to both strains. To address this issue, a sensitivity analysis (S1–S3 Tables) was performed after excluding those seronegative pairs (titer < 1:10 in matched specimens). The results of the sensitivity analysis strongly supported the agreement of antibody titers was mainly driven by the seropositive pairs. As the guideline for interpreting ICC values are inflexible, Cohen’s Kappa and Pearson’s correlation coefficient were also used as the metric for comparison. The results of these tests were in accordance with the ICC analysis (S4 and S5 Tables).

This study is also the first to use longitudinal blood specimens harvested from blood donation archives to study seroconversion rates of influenza. Age-standardised seroconversion rates under the conventional definition inferred from paired EDTA-plasma were consistent with those in paired sera from a separate serological study. Even if the alternative definition was adopted or subjects were stratified into age groups, the age-standardised seroconversion rates remain comparable. Particularly, there was no statistically significant difference in seroconversion rates between EDTA-plasma and serum for the two epidemics (S6 Table).

As such, the comparative analysis of seroconversion rates further provided evidence on the validity of EDTA-plasma serology. If blood donation archives can be synergistically linked with medical records containing personal history of influenza vaccination and diagnoses, they would provide a valuable source of longitudinal specimens for a wide range of influenza studies. For example, blood donation archives can be used for retrospective seroepidemiologic studies to assess the historical burden of influenza, especially in populations where the archived specimens are stored for very long periods. In the event of an influenza pandemic, substantial antigenic drift in seasonal influenza or vaccine mismatch, the archived specimens could be used to provide a rapid assessment of immunity among the adult populations. For epidemic serosurveillance, the archived specimens could serve as the pre-epidemic specimens for longitudinal sampling with specimens that will be collected from repeated donors as the epidemic unfolds for timely and accurate estimation of infection attack rate and severity [3]. To study antibody kinetics (e.g. antibody boosting after exposure to influenza, antibody waning, etc), longitudinal specimens from repeated donors in donation archives can be used for lookback studies on temporal changes in their antibody titers over a long period of time [37]. Serological data derived from blood donation archives would complement the data obtained from clinical or laboratory-confirmed infections because subjects in the archives would include subclinical cases because of their lack or mildness of symptoms [3].

Our study has several important limitations. First, we have only tested A/H1N1 and A/H3N2 for comparative analyses. In particular, we have not validated EDTA-plasma serology for influenza B. To establish the general validity of plasma influenza serology, the next phase of the validation should employ a much more comprehensive panel of test strains.

Second, compared with HI assay, enzyme-linked immunosorbent assay (ELISA) could yield unbiased results because it does not require the pre-treatment of blood samples or use of red blood cells, which might pose a problem for serology against some virus strains [38]. Ng et al. [39] reported that participants who tested negative in the HI assay had a high level of full-length hemagglutinin antibodies measured by ELISA. If ELISA is applied to detect antibody titer in serum and EDTA-plasma, it could provide more information on the relationship of antibody titers in matched pairs, especially for those specimens that were negative in the HI assay.

Third, there were sample collection time gaps between the paired sera in Kwok et al. and the paired EDTA-plasma from blood donation archives: around 6 months between the pre-season collection of sera and EDTA-plasma for the A/H3N2 epidemic in 2010; and around 3 months between the post-season collection of sera and EDTA-plasma for the A/H1N1 epidemic in 2011 (Fig 1). As virologic surveillance indicated that influenza activity was at negligible levels during these gaps, the impact of different collection times on the inference of seroconversion rates should be small.

When estimating the seroconversion rates from the blood donation archives, however, we did not know the influenza vaccination or infection history of the blood donors. One hundred and thirteen of four hundred and twenty participants in Kwok et al. were documented as having received a vaccination during the study period, and most vaccinated subjects were elderly aged 65 years or above. As serological assay cannot distinguish antibody response induced by vaccine from the response to natural infection, those vaccinated persons were excluded from the estimation of seroconversion rates. The vaccine coverage for seasonal influenza vaccine in adults aged 16–65 years in Hong Kong is generally lower than the coverage in adults aged 65 years or above as the latter are regarded as the high-risk group in vaccination programmes [40, 41]. Therefore, the effect of vaccination coverage might be very small in individuals aged 16–65 years. The set of blood donors with paired EDTA-plasma should be comparable with those individuals aged 16–65 years with paired sera.

In summary, our study provides proof-of-concept that blood donation archives could be leveraged as a valuable source of longitudinal blood specimens for the surveillance, control and risk assessment of influenza. These findings could also substantially help the Consortium for the Standardization of Influenza Seroepidemiology (CONSISE) [42] to develop best practices and standardize seroepidemiologic methods.

The general validity of plasma influenza serology could be embedded into a longitudinal seroepidemiologic study that collects serial serum and plasma specimens from a cohort including children, adults and elderly over several influenza seasons with monitoring of influenza vaccinations and laboratory-confirmed infections. Not only will these serological data be crucial for establishing the general validity of plasma influenza serology, but they will also provide a golden opportunity for studying antigenic seniority and the impact of infection and vaccination on the antibody landscape [43].

## Supporting information

S1 File This is the protocol of HI and MN assays. (PDF)

S1 Table This is marginal homogeneity of antibody titer distributions in matched serum and EDTA-plasma. (PDF)

S2 Table This is ICC values of HI titers in pairs after excluding seronegative pairs under the definition of absolute agreement versus consistency. (PDF)

S3 Table This is ICC values of MN titers in pairs after excluding seronegative pairs under the definition of absolute agreement versus consistency. (PDF)

S4 Table This is Cohen’s kappa of HI and MN titers between matched serum/EDTA-plasma specimens. (PDF)

S5 Table This is Pearson’s correlation coefficient of HI and MN titers between matched serum/EDTA-plasma specimens. (PDF)

S6 Table This is the comparative analyses of seroconversion rates between paired sera and EDTA-plasma in age groups. (PDF)

S1 Data (XLSX)

S2 Data (XLSX)

S3 Data (XLSX)

S4 Data (DOCX)
