# Supplementary material for: Harnessing the potential of blood donation archives for influenza surveillance and control
Source: PLoS One. 2020 May 29;15(5):e0233605. doi: 10.1371/journal.pone.0233605 (PMC7259782; doi:10.1371/journal.pone.0233605)
Supplement: S1 File — (PDF) [file pone.0233605.s001.pdf]

Serological assays

Hemagglutination inhibition (HI) assay. The protocol of HI assay was in accordance with Manual for the Laboratory Diagnosis and Virological Surveillance of Influenza by World Health Organization (WHO). HI assay was performed in V-bottom 96-well microtiter plates (Greiner, Germany) and each specimen was tested in duplicate. To remove non-specific inhibitors of HA, serum/EDTA-plasma were treated with receptor-destroying enzyme (RDE, Denka Seiken, Japan) at ratio 1:3 and incubated at 37°C for 16-18 hours. The remaining RDE enzyme was heat inactivated at 56°C for 30 mins, followed by adding 6 volumes of physiological saline (0.85% NaCl) to achieve the initial dilution 1:10. Further, RDE-treated serum/EDTA-plasma was heme-adsorbed with packed Turkey red blood cells (TRBC, Lampire, US) at 4°C for 1 hour. Serial two-fold dilutions of RDE-treated serum/EDTA-plasma were mixed with standardized A/H1N1/California/7/2009 (pandemic A/H1N1, WHO) or A/H3N2/Victoria/210/2009 (seasonal A/H3N2, WHO) antigen containing 4 hemagglutination units. After 1-hour incubation at room temperature, 0.5% TRBCs were added to each well. Plates were incubated at room temperature for 30 mins. The HI titer was defined as the reciprocal of the highest dilution of RDE-treated serum/EDTA-plasma that completely inhibited hemagglutination. Standardized influenza antigen back titration, positive and negative controls were included in each assay.

Microneutralization (MN) assay. The protocol of microneutralization assay was adapted from Manual for the Laboratory Diagnosis and Virological Surveillance of Influenza by World Health Organization (WHO). Influenza virus (A/H1N1/California/7/2009, pandemic A/H1N1 and A/H3N2/Victoria/210/2009, seasonal A/H3N2) was propagated in Madin-Darby Canine Kidney (MDCK) cells. MDCK

cell monolayers were grown in 96-well tissue culture plate (TPP, Switzerland) and each well contains around $1.5x10^4$ cells. Serum/EDTA-plasma were heat inactivated at 56°C for 30 mins. Serial two-fold dilutions of serum/EDTA-plasma beginning at 1:5 were mixed with 100x 50% tissue culture infectious dose ($TCID_{50}$) of virus for 2-hour incubation at 37°C with 5% CO_2. Next the serum-virus mixture was transferred to quadruplicate wells of MDCK cell plates for 1-hour infection at 37°C with 5% CO_2. After 1-hour infection, the serum-virus mixture was removed and the Minimal Essential Medium (MEM, GIBCO, US) supplemented with 10mM HEPES (GIBCO, US), Penicillin/Streptomycin (GIBCO, US) and 1μg/ml TPCK-trypsin (Sigma-Aldrich, US) was added. Incubated MDCK cell plates at 37°C with 5% CO_2 and observed the cytopathic effect (CPE) under an inverted microscope at day 3. The MN titers were defined as the reciprocal of the highest dilution of sera that exhibited CPE in ≤ 2 wells (50% neutralization). $TCID_{50}$ virus back titration, positive and negative controls were included in each assay.
