# Supplementary material for: Harnessing the potential of blood donation archives for influenza surveillance and control
Source: PLoS One. 2020 May 29;15(5):e0233605. doi: 10.1371/journal.pone.0233605 (PMC7259782; doi:10.1371/journal.pone.0233605)
Supplement: S1 Table — (PDF) [file pone.0233605.s002.pdf]

S1 Table. Marginal homogeneity of antibody titer distributions in matched serum and EDTA-plasma

Marginal Homogeneity of titer distribution by Stuart Maxwell Test									
All pairs					After excluding seronegative pairs (titer < 1:10)				
Age(N)	A/H1N1		A/H3N2		Age	A/H1N1		A/H3N2	
	HI	MN	HI	MN		HI	MN	HI	MN
16-19(23)	0.43	0.91	0.14	0.65	16-19	0.54	0.91	0.24	0.76
20-29(159)	<0.05	0.34	<0.05	0.35	20-29	<0.05	0.44	<0.05	0.49
30-39(165)	<0.05	0.95	<0.05	0.44	30-39	<0.05	0.99	<0.05	0.74
40-49(169)	<0.05	0.69	<0.05	0.28	40-49	<0.05	0.69	<0.05	0.33
50-69(93)	<0.05	0.45	<0.05	0.58	50-69	<0.05	0.62	0.18	0.53
Total	<0.05	0.52	<0.05	0.09	Total	<0.05	0.64	<0.05	0.07
