# Supplementary material for: Harnessing the potential of blood donation archives for influenza surveillance and control
Source: PLoS One. 2020 May 29;15(5):e0233605. doi: 10.1371/journal.pone.0233605 (PMC7259782; doi:10.1371/journal.pone.0233605)
Supplement: S2 Table — (PDF) [file pone.0233605.s003.pdf]

S2 Table. ICC values of HI titers in pairs after excluding seronegative pairs under the definition of absolute agreement versus consistency

ICC analysis of HI titers in pairs after excluding seronegative pairs				
Age (N)	A/H1N1		A/H3N2	
	Absolute agreement	Consistency	Absolute agreement	Consistency
16-19 (23)	0.84 (0.65-0.94)	0.86 (0.69-0.95)	0.92 (0.87-0.95)	0.96 (0.91-0.98)
20-29 (159)	0.84 (0.76-0.89)	0.89 0.83-0.93)	0.76 (0.67-0.82)	0.85 (0.78-0.89)
30-39 (165)	0.54 (0.38-0.71)	0.65 (0.49-0.8)	0.67 (0.56-0.76)	0.77 (0.65-0.85)
40-49 (169)	0.63 (0.46-0.75)	0.72 (0.55-0.82)	0.74 (0.62-0.84)	0.85 (0.76-0.91)
50-69 (93)	0.55 (0.38-0.69)	0.68 (0.5-0.79)	0.69 (0.51-0.85)	0.8 (0.63-0.9)
Total	0.72 (0.66-0.78)	0.8 (0.75-0.85)	0.76 (0.71-0.8)	0.85 (0.81-0.88)

*Seronegative pairs are those pairs that titers < 1:10 in serum and EDTA-plasma.
