# Supplementary material for: Harnessing the potential of blood donation archives for influenza surveillance and control
Source: PLoS One. 2020 May 29;15(5):e0233605. doi: 10.1371/journal.pone.0233605 (PMC7259782; doi:10.1371/journal.pone.0233605)
Supplement: S4 Table — (PDF) [file pone.0233605.s005.pdf]

S4 Table. Cohen's kappa of HI and MN titers between matched serum/EDTA-plasma specimens

Age (N)	Cohen's kappa (95%CI)			
	A/H1N1		A/H3N2	
	HI	MN	HI	MN
16-19 (23)	0.82 (0.68-0.96)	0.99 (0.96-1.0)	0.92 (0.89-0.96)	0.96 (0.92-1.0)
20-29 (159)	0.85 (0.79-0.91)	0.95 (0.93-0.97)	0.79 (0.73-0.85)	0.96 (0.95-0.98)
30-39 (165)	0.59 (0.44-0.74)	0.93 (0.89-0.98)	0.74 (0.65-0.82)	0.93 (0.89-0.97)
40-49 (169)	0.68 (0.56-0.80)	0.95 (0.92-0.97)	0.77 (0.68-0.86)	0.96 (0.94-0.98)
50-69 (93)	0.60 (0.45-0.75)	0.91 (0.82-0.99)	0.75 (0.63-0.87)	0.92 (0.88-0.97)
Total	0.75 (0.70-0.80)	0.95 (0.93-0.96)	0.80 (0.76-0.83)	0.95 (0.94-0.97)
