# Supplementary material for: Harnessing the potential of blood donation archives for influenza surveillance and control
Source: PLoS One. 2020 May 29;15(5):e0233605. doi: 10.1371/journal.pone.0233605 (PMC7259782; doi:10.1371/journal.pone.0233605)
Supplement: S5 Table — (PDF) [file pone.0233605.s006.pdf]

S5 Table. Pearson's correlation coefficient of HI and MN titers between matched serum/EDTA-plasma specimens

Age (N)	Pearson's correlation coefficient (95% CI)			
	A/H1N1		A/H3N2	
	HI	MN	HI	MN
16-19 (23)	0.87 (0.71-0.94)	0.99 (0.97-1.0)	0.96 (0.90-0.98)	0.97 (0.94-0.99)
20-29 (159)	0.90 (0.86-0.92)	0.95 (0.93-0.96)	0.86 (0.82-0.90)	0.96 (0.95-0.97)
30-39 (165)	0.71 (0.62-0.78)	0.94 (0.92-0.96)	0.81 (0.75-0.86)	0.94 (0.92-0.95)
40-49 (169)	0.76 (0.69-0.82)	0.95 (0.93-0.96)	0.86 (0.81-0.89)	0.96 (0.94-0.97)
50-69 (93)	0.72 (0.61-0.81)	0.91 (0.86-0.94)	0.82 (0.75-0.88)	0.94 (0.91-0.96)
Total	0.82 (0.80-0.85)	0.95 (0.94-0.95)	0.86 (0.84-0.88)	0.95 (0.95-0.96)
