# Supplementary material for: Harnessing the potential of blood donation archives for influenza surveillance and control
Source: PLoS One. 2020 May 29;15(5):e0233605. doi: 10.1371/journal.pone.0233605 (PMC7259782; doi:10.1371/journal.pone.0233605)
Supplement: S6 Table — (PDF) [file pone.0233605.s007.pdf]

S6 Table. The comparative analyses of seroconversion rates between paired sera and EDTA-plasma in age groups

Age group	A/H3N2		A/H1N1	
	**1:40***	**1:20#**	**1:40***	**1:20#**
**16-45y**	0.025 (-0.032-0.082)	0.053 (-0.007-0.112)	0.026 (-0.134-0.081)	0.015 (-0.123-0.092)
**46-65y**	0.058 (-0.019-0.13)	0.061 (-0.017-0.14)	0.12 (-0.078-0.102)	0.013 (-0.079-0.105)
**Total**	0.023 (-0.021-0.067)	0.042 (-0.005-0.088)	0.004 (-0.068-0.058)	0.0001 (-0.064-0.064)

*conventional definition of seroconversion: 4-fold rise and the latter titer is ≥ 1:40;
#alternative definition of seroconversion: 4-fold rise and the latter titer can be 1:20.
