# Supplementary material for: Harnessing the potential of blood donation archives for influenza surveillance and control
Source: PLoS One. 2020 May 29;15(5):e0233605. doi: 10.1371/journal.pone.0233605 (PMC7259782; doi:10.1371/journal.pone.0233605)
Supplement: S4 Data — (DOCX) [file pone.0233605.s011.docx]

1. The excel file “S1_data_titercomp_ver2.xlsx” summarized the HI and MN titers of 609 matched serum/EDTA-plasma specimens against A/H1N1/California/7/2009 and A/H3N2/Victoria/210/2009.

| **Column** | **Explanation** | **Notes** |
| --- | --- | --- |
| H1_HI_serum | A/H1N1 HI titer of serum |  |
| H1_MN_serum | A/H1N1 MN titer of serum |  |
| H3_HI_serum | A/H3N2 HI titer of serum |  |
| H3_MN_serum | A/H3N2 HI titer of serum |  |
| H1_HI_plasma | A/H1N1 HI titer of plasma |  |
| H1_MN_plasma | A/H1N1 MN titer of plasma |  |
| H3_HI_plasma | A/H3N2 HI titer of plasma |  |
| H3_MN_plasma | A/H3N2 MN titer of plasma |  |
| Birth_year | Year of birth |  |
| Donation_yearmon | Month/Year of donation |  |
| age | Donor age | age = year of donation – year of birth |
| agegrp | Donor age group |  |

1. The excel file “S2_data_H3_ver2.xlsx” summarized the HI titer of 376 paired EDTA-plasma specimens against A/H3N2/Victoria/210/2009.

| **Column** | **Explanation** | **Notes** |
| --- | --- | --- |
| HI_preH3 | HI titer in Jul 2010 |  |
| Donation_preH3_yearmon | Donation in Jul 2010 |  |
| HI_postH3 | HI titer in Dec 2010 |  |
| Donation_postH3_yearmon | Donation in Dec 2010 |  |
| Birth_year | Year of birth |  |
| age | Donor age | age = 2010 – year of birth |
| agegrp | Donor age group |  |

1. The excel file “S3_data_H1_ver2.xlsx” summarized the HI titer of 392 paired EDTA-plasma specimens against A/H1N1/California/7/2009.

| **Column** | **Explanation** | **Notes** |
| --- | --- | --- |
| HI_preH1 | HI titer in Dec 2010 |  |
| Donation_preH1_yearmon | Donation in Dec 2010 |  |
| HI_postH1 | HI titer in Apr 2011 |  |
| Donation_postH1_yearmon | Donation in Apr 2011 |  |
| Birth_year | Year of birth |  |
| age | Donor age | age = 2010 – year of birth.  Donors aged 55-56 were grouped into the 56-65 age group to ensure the sub-group sample size is sufficient for age standardization. |
| agegrp | Donor age group |  |
